# Physical Activity and Mental Toughness as Antecedents of Academic Burnout among School Students: A Latent Profile Approach

**DOI:** 10.3390/ijerph16112024

**Published:** 2019-06-06

**Authors:** Peggy Cheung, Chunxiao Li

**Affiliations:** 1 Department of Health and Physical Education, The Education University of Hong Kong, Hong Kong, China; cheungpy@eduhk.hk; 2 National Institute of Education, Nanyang Technological University, Singapore 637616, Singapore

**Keywords:** school burnout, mental toughness, physical activity, adolescence, person-oriented approach

## Abstract

Background: The purpose of the current survey-based study was to identify patterns of academic burnout and their antecedents among secondary school students. Methods: Secondary school students (*n* = 1209) completed a survey measuring academic burnout, physical activity, and mental toughness. Results: Using latent profile analyses, three burnout profiles were identified: a “moderately engaged group” (*n* = 699; 57.8%), a “burnout group” (*n* = 389; 32.2%), and a “well-functioning group” (*n* = 121; 10.0%). Group comparisons revealed that the “well-functioning group” reported significantly higher levels of physical activity and mental toughness than the other two groups. Conclusions: These findings suggest that lack of physical activity and mental toughness are potential risk factors for academic burnout. These findings may inform the development of intervention programs for academic burnout.

## 1. Introduction

Burnout has been widely studied in professional work domains [1]. Job burnout has important associated psychological consequences, including an increased risk of depression [2], lower self-esteem [3], and a higher suicide risk [4]. Several contemporary studies have reported burnout among university and school students [5,6]. In a review of 16 studies, a marked incidence of school-related or academic burnout was observed [7]. Previous studies also identified an increased risk of academic burnout in recent years [8,9,10]. Data reported from Finland revealed that 10–15% of adolescents aged 14–16 years suffered from school burnout [5]. Similar to job burnout, academic burnout is reported to exert negative effects on adolescents’ mental health, including anxiety, depression, and suicidal ideation [11,12,13]. 

Research into job burnout has traditionally treated the phenomenon as a stress-related syndrome, characterized by symptoms of emotional exhaustion, cynicism or depersonalization, and lack of personal accomplishment [14]. Similarly, academic burnout typically refers to tiredness resulting from academic demands (emotional exhaustion), having a pessimistic outlook and lack of interest toward academic tasks (cynicism), and a feeling of incompetence as a student (academic inefficacy or reduced efficacy) [6]. As the concept of academic burnout was borrowed from job burnout [6], it is expected that they share similar constructs. Moreover, while job burnout usually ties to the work domain, academic burnout is mainly concerning the school setting [6,14].

Various approaches have been used for examining academic burnout, including variable-oriented and person-oriented approaches. The variable-oriented approach has been widely used in most empirical work on burnout, and burnout symptoms (group mean burnout scores) have typically been taken as the unit of analysis. This approach is primarily concerned with the relations between the variables studied [15]. From a person-oriented perspective, burnout is typically investigated at a within-person level, and the individual is taken as the unit of analysis [16,17]. The fundamental difference between these two approaches is that the former approach aims at investigating the relations between variables, whereas the latter one focuses on examining how variables group within individuals [17,18]. This latter approach allows the examination of the burnout syndrome and its development over time. Such methods can also be used to identify potential types of burnout symptoms and explore the distinction between burnout and other wellbeing-related variables on an individual basis [18].

The negative consequences of academic burnout for mental health highlight the need for researchers to explore the factors associated with the syndrome. The present research focused on two potential predictors of academic burnout: physical activity and mental toughness. Physical activity is thought to exert a mental health risk-reducing effect and has been reported to be associated with diminished levels of burnout symptoms in a cross-sectional survey with 177 adults [19]. In a meta-analysis of four longitudinal survey studies, moderate relationships between physical activity and burnout were observed [20]. However, these studies focused on emotional exhaustion in an occupational population, and little is known on the association between physical activity and burnout among young people. On the basis of its possible psychological and physiological mechanisms, it may be reasonable to expect that physical activity reduces the risk of academic burnout among adolescents.

Mental toughness is a concept related to the ability to remain determined, focused, confident, and in control under stress and pressure [21,22] and has been widely examined in sports psychology [23,24]. A recent cross-sectional study reported that an increase in burnout symptoms was observed among peers with low levels of mental toughness, although this finding was confined to students with high stress levels [25]. In another cross-sectional study of 207 medical students, participants with higher mental toughness scores reported fewer burnout symptoms, irrespective of their stress levels [26]. With the positive findings regarding its association with burnout, mental toughness is likely to provide a possible buffer to academic burnout. Using a person-oriented approach may further increase our understanding of the association between these factors.

Academic performance is considered a high priority in many countries [27], and understanding the potential antecedents of adolescent academic burnout may help practitioners formulate effective intervention programs. Given this background and the important gaps in the literature, the current study had two main aims: (a) to identify the profiles of academic burnout using a person-oriented analytic approach and (b) to examine antecedents of burnout profiles (i.e., physical activity and mental toughness). On the basis of previous findings (e.g., [28,29]), we hypothesized that multiple profiles of academic burnout could be identified (Hypothesis 1). Moreover, physical activity and mental toughness levels were hypothesized to correspond with the burnout profiles identified (e.g., [25,26]; Hypothesis 2).

## 2. Materials and Methods

### 2.1. Participants

In Hong Kong, it is compulsory for every child to receive six years of primary education and three years of secondary education (six years of secondary education in total). It is always important for students to attain high school performance in order to be admitted to top primary and secondary schools. In the last year of secondary school (Form 6), the students take exams leading to Hong Kong Diploma of Secondary Education (HKDSE). The HKDSE result will be used for continuing education in higher education institutions. To be eligible for participation in this survey, participants were required to: (a) be at least 12 years old, (b) be able to answer the survey form, and (c) be a secondary school student. A sample of Chinese students (*n* = 1209) was recruited from five secondary schools in Hong Kong through convenience sampling. Of the participants, 728 (60.2%) were male, and 481 (39.8%) were female. The participants had a mean age of 14.85 (*SD* = 1.78). The participants were from different school years, namely, Form 1 (*n* = 272, 22.5%), Form 2 (*n* = 248, 20.5%), Form 3 (*n* = 240, 19.9%), and Form 5 (*n* = 297, 24.6%).

### 2.2. Measures

#### 2.2.1. Physical Activity

The Physical Activity Questionnaire for Older Children (PAQ-C) [30], a self-administered scale designed for children and adolescents aged 8 to 14 years, was applied to assess participants’ physical activity level. Recent validation research supported the use of the scale among Chinese children [31]. The scale is designed to obtain a general estimate of physical activity levels over the last seven days. A moderate to strong correlation has been reported between PAQ-C scores and data obtained through accelerometers [30,31]. The questionnaire consists of nine items (e.g., “Number of sports participated in after school over last week”). The participants scored the items using a 5-point scale (1 = “very low”, 5 = “very high”). The average score for all items was computed for further analyses. 

#### 2.2.2. Mental Toughness

The Mental Toughness Index [32] was used to measure participants’ mental toughness. The reliability of validity of the Chinese version of the scale has been confirmed among Chinese students [33]. The scale consists of eight items (e.g., “I consistently overcome adversity”). The participants provided responses to scale items using a 7-point Likert scale (1 = “false, 100% of the time,” 7 = “true, 100% of the time”). An average scale score was computed for further analyses. A higher mean score represents a greater level of mental toughness.

#### 2.2.3. Academic Burnout

The Maslach Burnout Inventory-Student Survey (MBI-SS) [6] was employed to assess participants’ academic burnout. The psychometric properties of the Chinese translated scale have been supported among students in China [34]. Three dimensions of academic burnout are evaluated using this scale: emotional exhaustion (five items; e.g., “I feel used up at the end of a day in school”), cynicism (four items; e.g., “I doubt the significance of my studies”), and academic inefficacy (six items; e.g., “In my opinion, I am a good student”). Participants scored the items on a 7-point scale (0 = “never”, 6 = “always”). The academic inefficacy subscale was reversely coded. A summed score for each subscale was computed for subsequent analyses. A higher subscale score indicates a higher level of burnout. 

### 2.3. Procedures

Upon receiving ethical approval from the University Human Ethics Research Committee (no. 2017-2018-0261), school principals were contacted via email to invite their students to participate in this survey study. Participants’ and their parents’ written informed consent was obtained before the survey. The survey forms were then administered to the participants in a quiet classroom, which was supervised by a research assistant together with a school teacher. The participants were instructed by the researcher to provide accurate responses. It took each participant approximately 20 minutes to complete the whole survey. The participants did not receive any incentive for their participation. 

### 2.4. Data Analyses

Means and standard deviations of the scale scores were computed. Reliability tests were used to determine the internal reliability of each subscale/scale, and a Cronbach’s alpha (*α*) value larger than 0.70 was considered acceptable [35]. In addition, zero-order correlation tests were conducted to examine the correlations among demographic and major study variables, including age, gender, physical activity, mental toughness, and academic burnout. These preliminary analyses were conducted using IBM SPSS Statistics 25 (IBM Corp., Armonk, NY). 

A series of confirmatory factor analyses (CFAs) were then conducted to examine the factorial validity of each of the scales used, and the robust maximum likelihood estimator (SB*χ*^2^) method was applied. Several fit indices were used to determine the model fit, including the comparative fit index (CFI), the Tucker Lewis index (TLI), and the root-mean-square error of approximation (RMSEA). A value of CFI/TLI greater than 0.90 and a value of RMSEA lower than 0.08 indicate an adequate model fit [36]. Following CFAs, a series of latent profile analyses (LPAs) were employed to identify participants’ academic burnout profiles based on their responses to the MBI-SS. Models of one to five profiles were specified, and the robust maximum likelihood estimator was used. The number of starting values and the maximum number of iterations for optimization were increased to avoid local likelihood maxima (STARTS = 5000 1000, STITERATIONS = 60). Participants’ age and gender were not entered as covariates in the models, because these two variables had only a mild association with some of the major study variables (for details, see the Results section). Moreover, in research settings, LPAs are typically conducted without including covariates (i.e., unconditional LPA; [37]).

Several criterion indices were used to determine the optimal number of profiles [37]: the Bayesian information criterion (BIC), the sample-size-adjusted BIC (ABIC), the likelihood ratio test (LRT), and the bootstrap likelihood ratio test (BLRT), as well as entropy, average posterior probability, and number of cases per profile. Profiles with lower BIC and ABIC values are typically preferred. A *p* value smaller than 0.05 suggests retaining the current model (*k* profile) in comparison to the model with one fewer profiles (*k* − 1). An entropy value greater than 0.60 and an average posterior probability greater than 0.70 are generally considered adequate [38,39]. Higher values of entropy and average posterior probability indicate higher quality of profile classification. Models with a profile that has less than 5% of the cases are not considered meaningful, so were discarded [40]. The interpretability of the results was also considered to determine the optimal number of profiles. A Wald chi-square test using AUXILIARY function was used to compare mean differences (physical activity and mental toughness) across the identified profiles (see [41]). CFAs and LPAs were conducted using *M*plus 7.1 [42].

## 3. Results

### 3.1. Descriptive Statistics

Table 1 presents the descriptive statistics, internal consistency, and zero-order correlations of the study variables. Overall, the participants reported relatively low physical activity levels, a relatively high level of mental toughness, and moderate levels of academic burnout. The scales and subscales demonstrated good internal reliability (α = 0.85 to 0.94). In general, age and gender had moderate relationships with physical activity, mental toughness, and academic burnout subscales. Physical activity was positively associated with mental toughness (r = 0.28, *p* < 0.001). Physical activity and mental toughness were negatively related to academic burnout subscales (r = −0.09 to −0.46, *p* < 0.001).

### 3.2. CFAs

The one-factor measurement model of the Mental Toughness Inventory was supported: SB*χ*^2^(19) = 127.72, *p* < 0.001, CFI = 0.975, TLI = 0.964, RMSEA = 0.069 (0.058, 0.080). The data fit the unidimensional model of the Physical Activity Questionnaire for Older Children: SB*χ*^2^(26) = 75.72, *p* < 0.001, CFI = 0.987, TLI = 0.982, RMSEA = 0.040 (0.030, 0.050). The data also revealed an acceptable fit to the three-factor measurement model of the Maslach Burnout Inventory-Student Survey: SB*χ*^2^(82) = 395.99, *p* < 0.001, CFI = 0.957, TLI = 0.945, RMSEA = 0.056 (0.051, 0.067). 

### 3.3. LPAs

Table 2 presents the values of criterion indices. Obvious declines in BIC and ABIC values were observed until the model with five profiles. According to the LRT and BLRT results, the two-profile solution was better than the one-profile model. The LRT and BLRT results did not differentiate between models with 2–4 profiles. The entropy value of the two-profile model was lower than that of the models with 3–4 profiles. The three-profile solution had greater entropy and average posterior probability values than the four-profile solution. The three-profile solution was finally accepted by also considering the interpretability of the profile. 

**Figure 1:**
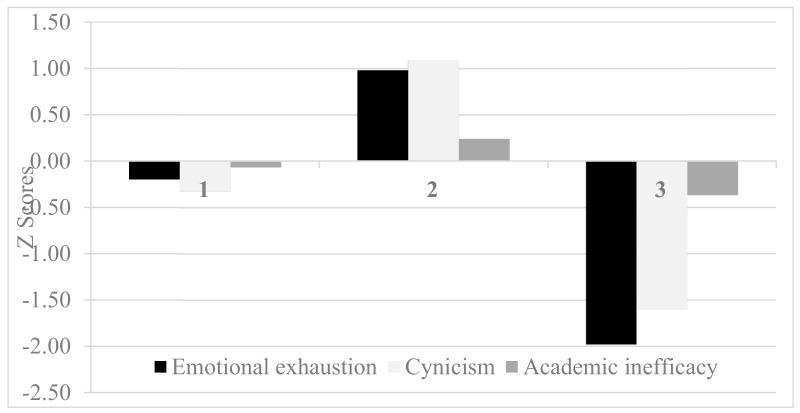
Academic burnout profiles based on standardized scores of each subscale. 1 = Profile 1 (moderately engaged group), 2 = Profile 2 (burnout group), 3 = Profile 3 (well-functioning group).

Figure 1 presents the Z scores of the academic burnout subscales of the accepted profiles. Profile 1 (n = 699; 57.8%) was a “moderately engaged group”, who scored moderately on all the academic burnout subscales. Profile 2 (n = 389; 32.2%) was a “burnout group”. This group reported the highest levels of emotional exhaustion and cynicism as well as a medium level of academic inefficacy. Profile 3 (n = 121; 10.0%) was a “well-functioning group”, reporting relatively low levels of academic burnout. Thus, the results supported Hypothesis 1.

### 3.4. Group Comparisons

Table 3 presents the results of mean comparisons of physical activity and mental toughness using the identified profiles. The three identified profiles (groups) differed significantly in terms of physical activity (overall Wald *χ*^2^ = 13.26, p = 0.001) and mental toughness (overall Wald *χ*^2^ = 55.18, p < 0.001). Profile 3 (“well-functioning group”) had higher mean scores for physical activity and mental toughness than the other two profiles (Wald *χ*^2^ = 9.41 to 37.91, p < 0.001). There was no group difference in physical activity between Profile 1 (“moderately engaged group”) and Profile 2 (“burnout group”): Wald *χ*^2^ = 1.23, p = 0.27. However, Profile 1 reported a higher mean score for mental toughness than Profile 2 (Wald *χ*^2^ = 28.22, p < 0.001). These results supported Hypothesis 2. 

## 4. Discussion

### 4.1. Identification of Burnout Profiles among Secondary School Students

The current study identified three academic burnout profiles among secondary school students in Hong Kong, supporting Hypothesis 1. Despite previous studies describing burnout as a homogenous concept with unitary and global terms [43], the present study examined the multidimensional structure of academic burnout, in accordance with previous studies reporting that constructing burnout profiles can reflect more consistent elements of burnout [44,45]. By adopting a person-oriented approach, burnout symptoms were investigated within individuals. The majority (57.8%) of adolescents fell into the “moderately engaged group” with moderate burnout scores for emotional exhaustion, cynicism, and academic inefficacy. Only a small portion (10.0%) of adolescents were classified into the “well-functioning group”, with lower scores for emotional exhaustion, cynicism, and academic inefficacy.

We classified 32.2% of adolescents into the “burnout group”, exhibiting a high level of burnout symptoms for emotional exhaustion and cynicism. However, academic inefficacy scores among this group were at a medium level. The burnout conditions of this group of adolescents were similar to those described among “persevering students” in Korea [44] and mainland China [46]. This group of adolescents were described as experiencing emotional and physical exhaustion in their school work but continued to be responsible and put effort into their study. In Asian cultures, hard work and effort are strongly emphasized over natural ability [47,48]. The cultural and educational systems in Asian countries, which typically place a high priority on academic performance, may have prompted adolescents to continue studying despite experiencing high levels of emotional exhaustion. Nevertheless, the high proportion of adolescents in the “burnout group” is alarming and highlights the need to take further measures to combat the issue. 

### 4.2. Physical Activity Participation and the Identified Burnout Profiles

The present study provided support for a difference in the level of physical activity among adolescents in various burnout conditions. Secondary school students with lower levels of burnout (“well-functioning group”: *M* = 2.44) tended to be involved in more physical activity than those with higher levels of burnout (“moderately engaged group”: *M* = 2.20; “burnout group”: *M* = 2.15). These findings supported Hypothesis 2. The finding was also in accord with previous studies reporting a negative association between burnout and physical activity among adult workers [49,50]. Similarly, in a study of 144 vocational students, it was reported that participants who fulfilled a basic physical activity recommendation (i.e., 75 min/week) exhibited lower levels of academic burnout [51].

The associations between specific burnout symptoms and physical activity have not been examined in detail in previous studies. Emotional exhaustion was the most frequently examined factor, and a positive association with physical activity was reported, while previous results concerning academic inefficacy and cynicism are less frequent and consistent [20]. By generating burnout profiles, the present study revealed a positive association between the combined burnout symptoms and physical activity. This approach is compelling for describing the physical activity patterns of adolescents in different burnout groups. 

The present findings can also contribute to future studies examining how physical activity can influence the development of burnout at an individual level. Physical activity participation appears to have a protective function, reducing the likelihood of adolescents experiencing academic burnout. Physical activity is a complex behavior, and the mechanisms underlying this association remain uncertain. According to previous research, this positive association could be related to psychological changes during physical activity involvement, such as behavioral distraction from stressful situations [52] and the development of self-efficacy [53]. Alternatively, it is possible that students with high levels of burnout would be less motivated to participate in any form of physical activity. The current finding of an association between physical activity and burnout in adolescents should be explored in future studies with designs that enable causality to be determined. 

### 4.3. Mental Toughness and the Identified Burnout Profiles

The present study also confirmed the association between adolescent mental toughness and different levels of academic burnout. Secondary school students with lower levels of burnout (“well-functioning group”: *M* = 5.50) had a higher level of mental toughness compared with those exhibiting higher levels of burnout (“moderately engaged group”: *M* = 5.03; “burnout group”: *M* = 4.67). Mental toughness scores among the “moderately engaged group” (*M* = 5.03) were higher than those in the “burnout group” (*M* = 4.67). The current results are in general agreement with the findings of previous studies reporting that mental toughness is associated with lower levels of burnout among vocational students [25,54], medical students [26], and junior athletes [55]. 

Individuals with high levels of mental toughness are characterized as those who persist during adverse or difficult circumstances, exhibiting the ability to cope with change and the simultaneous demands that occur during transitions and to see such processes as a challenge rather than a threat [56]. The current findings expanded previous research on burnout to secondary school students, providing new insights that may be useful for the development of intervention studies on academic burnout. Early adolescence is a distinct life stage that challenges young people with rapid physical and psychological changes. The characteristics of academic burnout occurring at this stage may differ from those among individuals in other age groups and occupations. The development of mental toughness also differs from that of athletes in sporting settings. Among adolescents, school is an ideal venue to provide effective environments for building mental toughness before entering adulthood. 

Our findings suggest increasing secondary school students’ physical activity and mental toughness levels may decrease their academic burnout. Thus, practitioners such as physical education teacher and health educators can develop school-based exercise and/or mental toughness programs to combat academic burnout. Despite the significant findings and implications outlined above, the current study involved several limitations that should be addressed. First, participants’ physical activity was measured using self-report questionnaires. Future studies should include objective measurements, such as accelerometer, to obtain additional physical activity data for analysis. Second, the cross-sectional study design did not enable conclusions about a casual direction of the observed effects. In addition, the mechanisms underlying the observed associations remain to be determined. 

## 5. Conclusions

In conclusion, the present study identified multidimensional profiles of academic burnout. The identified profiles broaden our understanding on academic burnout and highlight concerning levels of burnout among secondary school students. These findings suggest that low levels of physical activity and mental toughness are potential risk factors for academic burnout. Intervention study designs should be used in the future to explore the possible causal effects of physical activity and mental toughness on academic burnout, potentially leading to the prevention and alleviation of this negative psychological condition among school students. 

## Figures and Tables

**Table 1:** Descriptive statistics, internal reliability, and zero-order correlations of the study variables (*n* = 1209).

	1.	2.	3.	4.	5.	6.	7.
1. Age	-						
2. Gender	0.01	-					
3. Physical activity	−0.21 ***	−0.16 ***	-				
4. Mental toughness	−0.09 ***	−0.08 ***	0.28 ***	-			
5. Emotional exhaustion	0.09 **	0.08 ***	−0.12 ***	−0.23 ***	-		
6. Cynicism	0.11 ***	0.02	−0.09 ***	−0.23 ***	0.76 ***	-	
7. Academic inefficacy	0.06	−0.07 *	−0.15 ***	−0.46 ***	0.14 ***	0.21 ***	-
*α*	-	-	0.85	0.94	0.85	0.88	0.85
M	14.85	-	2.21	4.96	17.07	12.06	12.54
SD	1.78	-	0.72	1.05	5.83	5.39	6.33
Range	12–21	-	1–5	1–7	0–30	0–24	0–36

Note. * *p* < 0.05, ** *p* < 0.01, *** *p* < 0.001.

**Table 2:** Values for different model parameterizations (*n* = 1209).

Model	BIC	ABIC	LRT *p*-Value	BLRT *p*-Value	Entropy	APP	Group Size ≤ 5%
1 profile	23128.10	23109.04	-	-	-	-	0
2 profiles	22574.79	22543.02	<0.001	<0.001	0.66	0.90	0
3 profiles	22121.56	22077.09	<0.001	<0.001	0.82	0.92	0
4 profiles	21848.68	21913.00	<0.001	<0.001	0.80	0.90	0
5 profiles	21878.59	21778.79	<0.001	<0.001	0.83	0.92	1

Note. AIC: Akaike’s information criterion, BIC: Bayesian information criterion, ABIC: sample-size-adjusted BIC, LRT: likelihood ratio test, BLRT: bootstrapped likelihood ratio test, APP: average posterior probability.

**Table 3:** Descriptive Scores of physical activity and mental toughness by profile (*n* = 1209).

	Profile 1 (Moderately Engaged Group)	Profile 2 (Burnout Group)	Profile 3 (Well-Functioning Group)	Overall Wald *χ*^2^	Profile Comparisons
Physical activity	2.20 (0.97)	2.15 (1.39)	2.44 (2.43)	13.26 **	3 > 1, 2 ***, 1 = 2
Mental toughness	5.03 (1.32)	4.67 (1.88)	5.50 (4.28)	55.18 ***	3 > 1, 2 ***, 1 > 2 ***

Note. ** *p* < 0.01, *** *p* < 0.001.
